# Identification of Hub Genes in Idiopathic Pulmonary Fibrosis and Their Association with Lung Cancer by Bioinformatics Analysis

**DOI:** 10.3390/arm91050032

**Published:** 2023-10-12

**Authors:** Juan Manuel Velázquez-Enríquez, Itayetzi Reyes-Avendaño, Jovito Cesar Santos-Álvarez, Edilburga Reyes-Jiménez, Verónica Rocío Vásquez-Garzón, Rafael Baltiérrez-Hoyos

**Affiliations:** 1 Laboratorio de Fibrosis y Cáncer, Facultad de Medicina y Cirugía, Universidad Autónoma Benito Juárez de Oaxaca, Ex Hacienda de Aguilera S/N, Sur, San Felipe del Agua, Oaxaca 68020, Mexico; juanmanuelvela_enriquez@cecad-uabjo.mx (J.M.V.-E.); itayetzi.reyes94@cecad-uabjo.mx (I.R.-A.); jovitocesarsa@cecad-uabjo.mx (J.C.S.-Á.); edilreyesjimnez@cecad-uabjo.mx (E.R.-J.); vrvasquezga@conacyt.mx (V.R.V.-G.); 2 CONAHCYT-Facultad de Medicina y Cirugía, Universidad Autónoma Benito Juárez de Oaxaca, Ex Hacienda de Aguilera S/N, Sur, San Felipe del Agua, Oaxaca 68020, Mexico

**Keywords:** differentially expressed genes, idiopathic pulmonary fibrosis, lung cancer, meta-analysis, microarrays

## Abstract

**Highlights:**

**What are the main findings?**

- Identification of 1888 differentially expressed genes (DEGs) related to idiopathic pulmonary fibrosis (IPF), including 1105 upregulated and 783 downregulated genes.
- Discovery of 10 hub genes with high connectivity that may play a crucial role in the pathogenesis of IPF, with implications for potential diagnostic biomarkers and therapeutic targets.

**What is the implication of the main finding?**

- The study sheds light on the genetic landscape of IPF, uncovering potential key players in its development and progression.
- These identified hub genes have relevance beyond IPF, being expressed in lung cancer and associated with various stages of cancer progression, suggesting a link between IPF and lung cancer that could pave the way for improved diagnostics and therapies.

**Abstract:**

Background: Idiopathic pulmonary fibrosis (IPF) is a chronic, progressive, and irreversible disease with a high mortality rate worldwide. However, the etiology and pathogenesis of IPF have not yet been fully described. Moreover, lung cancer is a significant complication of IPF and is associated with increased mortality. Nevertheless, identifying common genes involved in developing IPF and its progression to lung cancer remains an unmet need. The present study aimed to identify hub genes related to the development of IPF by meta-analysis. In addition, we analyzed their expression and their relationship with patients’ progression in lung cancer. Method: Microarray datasets GSE24206, GSE21369, GSE110147, GSE72073, and GSE32539 were downloaded from Gene Expression Omnibus (GEO). Next, we conducted a series of bioinformatics analysis to explore possible hub genes in IPF and evaluated the expression of hub genes in lung cancer and their relationship with the progression of different stages of cancer. Results: A total of 1888 differentially expressed genes (DEGs) were identified, including 1105 upregulated and 783 downregulated genes. The 10 hub genes that exhibited a high degree of connectivity from the PPI network were identified. Analysis of the KEGG pathways showed that hub genes correlate with pathways such as the ECM–receptor interaction. Finally, we found that these hub genes are expressed in lung cancer and are associated with the progression of different stages of lung cancer. Conclusions: Based on the integration of GEO microarray datasets, the present study identified DEGs and hub genes that could play an essential role in the pathogenesis of IPF and its association with the development of lung cancer in these patients, which could be considered potential diagnostic biomarkers or therapeutic targets for the disease.

## 1. Introduction

Idiopathic pulmonary fibrosis (IPF) is a chronic disease of progressive and irreversible character. It is known as the most severe and common idiopathic interstitial pneumonia (IIP) [1,2]. To date, the pathogenesis of IPF has not been fully elucidated; however, one of the best-accepted hypotheses proposes that IPF is characterized by increased fibroblast proliferation, activation, and extracellular matrix (ECM) secretion and deposition as a result of persistent injury and inflammation in the lung, resulting in chronic fibrosis in the lung parenchyma, with progressive disease, a significant reduction in lung function, and culminating in respiratory failure and, unfortunately, death of the patient [1,3]. Reports have estimated an incidence of 3 to 9 cases per 100,000 inhabitants per year in European and North American countries. In contrast, data have estimated a lower incidence rate in East Asian and South American countries [4]. The incidence and prevalence of IPF increase considerably with advanced age, manifesting mainly in adults over 60 years of age, with a higher frequency in men [2,3]. IPF has a high mortality rate related to a life expectancy of 3–5 years after diagnosis, because IPF is usually diagnosed at an advanced stage of the disease and there is a scarcity of effective treatments. Two antifibrotic drugs, pirfenidone and nintedanib, are currently approved for the treatment of IPF; however, they cannot completely reverse the course of the disease [1,3,5]. According to international guidelines, the diagnosis of patients with suspected IPF should be made using a multidisciplinary approach. It is based on identifying a radiological and histopathological pattern characteristic of usual interstitial pneumonia (UIP) and should focus on excluding possible causes related to pulmonary fibrosis [6,7]. However, the diagnosis of IPF is usually made at an advanced stage of the disease, because during the early stages, patients present with an asymptomatic form of IPF, and a variety of potential diagnostic biomarkers for IPF are now available. However, they are not specific enough to differentiate IPF from other interstitial lung disease (ILDs); the guidelines for diagnosing IPF do not recommend using these biomarkers [8]. Therefore, the search for new biomarkers that help understand the main molecular alterations that favor the development of IPF is essential to facilitate an early diagnosis.

Several studies have identified potential genetic and environmental risk factors that promote the development of IPF [1,5]. Chronic exposure to environmental factors such as tobacco smoke, silica, metals, and wood dust has been associated with the development of IPF [2,3,5]. Recently, viral infections such as hepatitis C virus; human herpesvirus 8; Epstein–Barr virus; cytomegalovirus; and some coronaviruses, including severe acute respiratory syndrome coronavirus (SARS-CoV), Middle East respiratory syndrome coronavirus (MERS-CoV), and severe acute respiratory syndrome coronavirus 2 (SARS-CoV-2), have been implicated in the onset of IPF. Therefore, with the global pandemic of coronavirus disease 2019 (COVID-19), caused by SARS-CoV-2 infection, an increase in the prevalence of patients with pulmonary fibrosis is estimated, because patients who have recovered from COVID-19 are at increased risk of pulmonary complications, including the development of pulmonary fibrosis [5,9,10,11]. Therefore, we must prepare ourselves to understand the pathogenesis and find biomolecules for diagnosis and therapeutic targets for the possible increase in cases of pulmonary fibrosis.

IPF is associated with various symptoms, such as dyspnea, cough, weight loss, and chest discomfort or pain [3,12]. The main complications that a patient with IPF may suffer include depression, pulmonary hypertension, and lung cancer [2,13]. Recent research has shown that lung cancer is positioned as one of the main complications in patients with IPF, with LUAD (lung adenocarcinoma) and LUSC (lung squamous cell carcinoma) being the two most frequent types of lung cancer in these patients. The available statistics indicate that between 7% and 20% of patients have a high risk of developing lung cancer, which is associated with a poor prognosis and translates into increased mortality in these patients [14,15]. Therefore, there is a need to improve the understanding of the main mechanisms associated with the development of lung cancer in patients with IPF.

In recent decades, studies focused on the analysis of gene expression data have been widely used in biomedical research to identify differentially expressed genes (DEGs), establish potential biomarker candidates, and identify the main alterations at the molecular level that allow detailed elucidation of the main pathways involved in the development of different diseases [16,17]. The microarray datasets generated by gene expression analyses are stored in public databases such as Gene Expression Omnibus (GEO) [18].

In this regard, a considerable number of investigations have applied gene expression data analysis technology to identify hub DEGs in IPF to identify the central target genes involved in the onset and development of the disease, which has facilitated the elucidation of the main molecular pathways and the identification of potential diagnostic biomarkers for IPF [19,20,21,22,23]. However, it has been argued that there are inconsistencies in the results obtained in the different studies applying gene expression data analysis technology, mainly attributed to the small sample sizes used, the different statistical methods employed, and the different microarray platforms used to generate these gene expression datasets. Bearing this in mind, because these raw datasets are stored and available in public databases, integration and meta-analysis of these gene expression datasets is a useful tool to improve inconsistencies and obtain more reliable results [24,25].

Therefore, to identify DEGs, our study aimed to perform a meta-analysis of multiple microarray-generated lung tissue gene expression datasets from IPF patients and healthy controls. In addition, Gene Ontology (GO) enrichment analysis and Kyoto Encyclopedia of Genes and Genomes (KEGG) pathway enrichment analysis were performed to further interpret the DEGs. Additionally, a protein-protein interaction (PPI) network of the DEGs, significant modules, and hub genes of the PPI network was established. Additionally, miRNAs targeting identified hub genes were predicted. Additionally, we explored the association between hub genes and lung cancer progression. The results obtained in the present study provide information on the development of IPF at the molecular level and identify biomarker candidates for the diagnosis and treatment of IPF patients.

## 2. Materials and Methods

### 2.1. Ethical Statement

This study did not require ethical approval, because the data analyzed are freely available in public databases.

### 2.2. Selection and Inclusion Criteria for Gene Expression Microarray Data

To analyze gene expression profiles in IPF, independent gene expression microarray datasets related to IPF were retrieved and downloaded from the GEO database (https://www.ncbi.nlm.nih.gov/geo/, accessed on 4 July 2021). A search of publicly available gene expression datasets from 1 January 2010 through 30 June 2021 was performed using the keyword “idiopathic pulmonary fibrosis”. The search results were further narrowed as follows: series (type of entry), “expression profile by matrix” (type of study), and Homo sapiens (organism). The identified microarray datasets were reviewed, filtered, and selected according to our inclusion criteria: (1) human gene expression microarray data, (2) datasets using lung tissues for gene expression analysis, (3) complete gene expression data available (raw or normalized), and (4) the datasets included fibrotic and nonfibrotic lung tissues. Finally, five independent gene expression microarray datasets (GSE24206, GSE21369, GSE110147, GSE72073, and GSE32539) were obtained. The following information corresponds to each dataset retrieved: GEO accession ID, control sample size, IPF sample size, microarray platform, and raw gene expression data.

### 2.3. Identification of DEGs

Five gene expression microarray datasets of lung tissue from IPF patients (GSE24206, GSE21369, GSE110147, GSE72073, and GSE32539) were selected for gene expression meta-analysis using the ImaGEO platform (http://bioinfo.genyo.es/imageo/, accessed on 7 July 2021), a web-based application for integration and meta-analysis [26]. The ImaGEO platform performs a meta-analysis of gene expression data by applying the functions of the MetaDE R package. Each dataset was retrieved and loaded using the GEOID. We used the effect size combination method, specifically the random effects model (REM) and default settings for the integration of differential gene expression. REM is one of the most commonly used methods in meta-analysis for combining gene expression effect sizes, where the studies included in the analysis contain a random effect that may incorporate unknown between-study heterogeneities, mainly attributable to different platforms or different batches [26]. Therefore, DEGs with the strongest average effect across all datasets included in the study were identified. The Z value was calculated using the formula that compares the pooled effect with the variability or dispersion of the effects in the individual studies. Once the Z value was calculated, it was compared with a standard normal distribution (known as the Z table) to determine whether the pooled effect was statistically significant. Genes reporting an adjusted *p* < 0.05 and a Z value > 2.5 were identified as upregulated DEGs, while genes reporting an adjusted *p* < 0.05 and a Z value < −2.5 were identified as downregulated DEGs and were selected for further analysis.

### 2.4. GO Enrichment and KEGG Pathway Enrichment Analyses

GO analysis, including biological processes (BP), molecular function (MF), cellular components (CC), and KEGG pathway analysis for DEGs, were applied using the Database for Annotation, Visualization, and Integrated Discovery (DAVID) (https://david.ncifcrf.gov/, accessed on 11 July 2021) [27,28]. Gene set enrichment results with *p* < 0.05 were considered statistically significant. Proteins from the KEGG pathway analysis were loaded into ShinyGO v0.66 software (http://ge-lab.org/go/, accessed on 26 August 2021), and pathway diagrams were retrieved from the KEGG web server using the Bioconductor pathview package [29].

### 2.5. PPI Network Analysis

To explore the interaction networks of DEGs, an interaction network was constructed using the search tool for the retrieval of interacting genes/proteins (STRING) version 11 (https://string-db.org/, accessed on 10 July 2021) [30,31], which was used to observe the interaction and functional enrichment of DEGs. The network was constructed with a minimum required interaction score > 0.9. Visualization of the PPI network was performed using Cytoscape software [32]. The MCODE add-on of Cytoscape was used to select the significant modules of the PPI network, with the cutoff degree = 2, depth = 100, k-core = 2, and node score cutoff point = 0.2. The Cytoscape add-on cytoHubba was used to explore the hub genes of the PPI network using the MCC method.

### 2.6. Validation of the Hub Genes

The Fibrosis-Related Omnibus for Archives and Datasets (FibROAD) was used to confirm the validity and relevance of hub genes identified in IPF through meta-analysis. FibROAD (https://www.fibroad.org, accessed on 2 September 2022) is an open-access database that integrates evidence of fibrosis-associated disorders in multiple organs obtained from multiomic data, providing online validation of fibrosis-associated genes [33]. The GSE92592 [34] dataset corresponding to a lung tissue mRNA sequencing experiment of IPF samples (*n* = 20) and control samples (*n* = 19), available in FibroAD via DatasetID: SRP095361, was used to analyze the differential expression of hub genes. Fragments per kilobase of transcript per million mapped reads (FPKM) values of genes were analyzed individually. Statistical significance was assessed by two-tailed *t*-test analysis using GraphPad Prisma 8.0.1 software (GraphPad, San Diego, CA, USA), and a statistically significant difference was considered to exist when *p*-values were <0.05.

### 2.7. Analysis for miRNA Target Gene Prediction and miRNA-mRNA Network Construction

We used the miRWalk 3.0 (http://mirwalk.umm.uni-heidelberg.de/, accessed on 1 September 2021) database to predict the interaction between miRNAs and the hub genes identified in our study [35]. miRWalk 3.0 is a publicly available platform that hosts information on predicted and experimentally validated miRNA–target binding sites. Setting the configuration for the analysis to a 0.9 score, the target gene binding region was 3′ UTR, and intersections with other databases were established for miRDB. In addition, we used Cytoscape to generate a regulatory network between the miRNA and its target genes.

### 2.8. Analysis of Hub Gene Expression in Lung Cancer

To evaluate the expression of hub genes in LUAD and LUSC and their relationship with cancer progression, we used the UALCAN database (http://ualcan.path.uab.edu/, accessed on 7 April 2022), a comprehensive web-based tool, to analyze and visualize omics data in different cancer types and allowed users to concisely identify the gene expression of the mRNA of interest and assess its correlation with cancer progression [36]. In this study, the expression of hub genes in lung cancer samples (LUAD and LUSC) was analyzed and compared to that in normal tissue samples. A statistically significant difference was considered when *p*-values were <0.05.

## 3. Results

### 3.1. Processing of Microarray Datasets and Identification of DEGs in IPF

**Figure 1:**
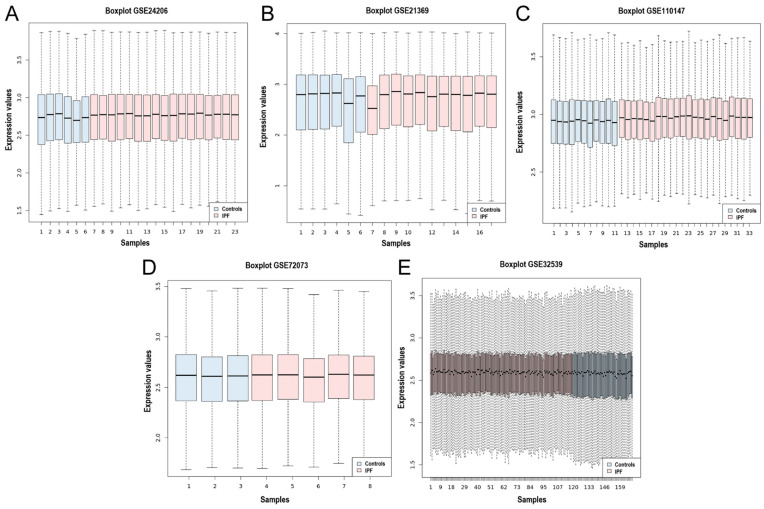
Box plot of the data normalization for the 5 datasets. Box plot of the data normalization for (**A**) GSE24206, (**B**) GSE21369, (**C**) GSE110147, (**D**) GSE72073, and (**E**) GSE32539. The *X*-axis represents the controls and IPF samples, while the *Y*-axis is the gene expression value.

Five gene expression microarray datasets (GSE24206, GSE21369, GSE110147, GSE72073, and GSE32539) were selected in which the transcriptome of lung tissue from IPF patients was compared to that of healthy controls. The datasets were obtained from the National Center for Biotechnology Information-Gene Expression Omnibus (NCBI-GEO). An overview of the datasets included in this meta-analysis is shown in Table 1. A total of 76 control samples and 174 IPF samples were integrated using the MetaDE R package. In addition, the homogeneity of the expression values of all the datasets was assessed, and the box plot indicates the measure of centrality of each of the datasets and shows the homogeneity in the expression values (Figure 1A–E).

**Figure 2:**
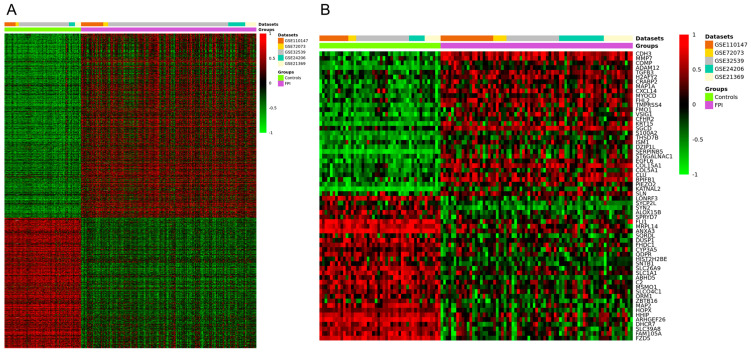
Clustering heatmap for the DEGs identified from the meta-analysis of datasets GSE24206, GSE21369, GSE110147, GSE72073, and GSE32539. (**A**) Clustering heatmap of the total DEGs. (**B**) Hierarchical clustering heatmap of the top 60 DEGs. Red indicates upregulated genes, and green indicates downregulated genes.

The meta-analysis identified a total of 1888 DEGs (Figure 2A), of which 1105 were upregulated (*p* < 0.05 and a Z value > 2.5) and 783 were downregulated (*p* < 0.05 and a Z value < −2.5). The heatmap of the top 60 upregulated and downregulated genes is shown in Figure 2B, and the full list of DEGs can be found in Table S1.

### 3.2. GO Enrichment and KEGG Pathway Analyses for DEGs in IPF

**Figure 3:**
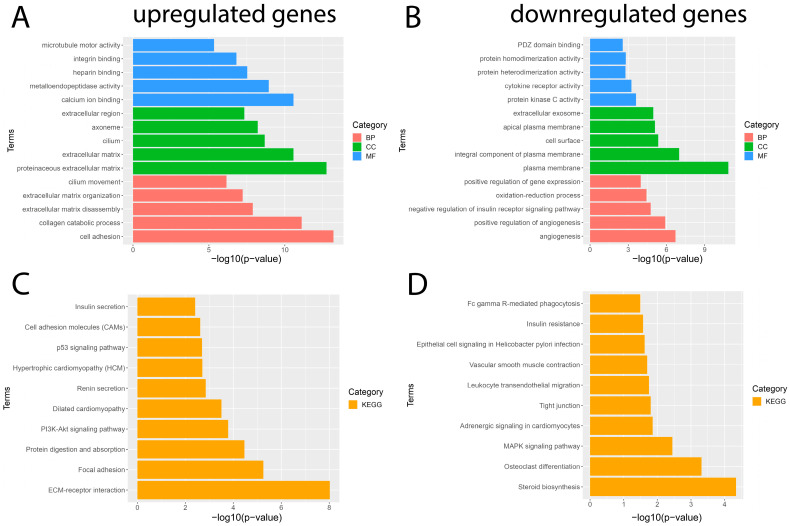
Results of GO and KEGG pathway enrichment analysis of DEGs. The five most important terms for each of the three categories of the GO analysis of the significantly (**A**) upregulated and (**B**) downregulated genes. The ten most important terms for the KEGG pathway enrichment analysis for (**C**) upregulated and (**D**) downregulated genes. Red represents a biological process (BP), blue represents molecular function (MF), green represents cellular component (CC), and orange represents the Kyoto Encyclopedia of Genes and Genomes (KEGG) pathway enrichment analysis.

To gain a better understanding of the main functions and mechanisms in which DEGs are involved, we performed GO enrichment and KEGG pathway analyses employing the DAVID platform with a significance value of *p* < 0.05. The results obtained from the GO analysis showed that the BP of the significantly upregulated genes were mainly enriched for cell adhesion and the collagen catabolic process; the significantly downregulated genes were mainly involved in angiogenesis and the positive regulation of angiogenesis. The MF of significantly upregulated genes was mainly enriched for calcium ion binding and metalloendopeptidase activity; significantly downregulated genes were involved in protein kinase C activity and cytokine receptor activity. The CC of significantly upregulated genes was mainly enriched for the proteinaceous extracellular matrix and extracellular matrix; significantly downregulated genes were mainly involved in the plasma membrane and plasma membrane integral component (Figure 3). In addition, the results obtained from the KEGG pathway enrichment analysis showed that the significantly upregulated genes were mainly enriched in pathways such as ECM–receptor interaction, focal adhesion, protein digestion, and uptake; the results obtained for the significantly downregulated genes showed that they were mainly enriched for the steroid biosynthesis pathway, osteoclast differentiation, and MAPK signaling pathway (Figure 3). The complete list of GO and KEGG pathway analyses can be found in Tables S2 and S3.

### 3.3. PPI Network and Identification of Hub Genes

**Figure 4:**
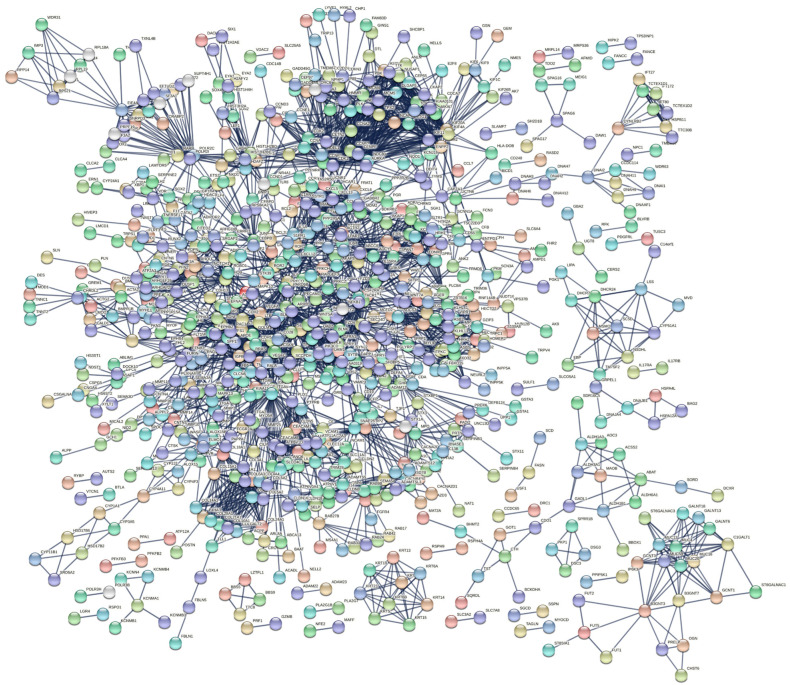
PPI network of DEGs constructed using STRING software. The DEGs were combined to construct a regulatory network using STRING version 11 software to visualize the interaction and functional enrichment with tests such as the importance of network edges, and the active interaction sources were Text mining, Experiments, Database, Co−expression, Neighborhood, Gene Fusion, and Co-occurrence, with a minimum interaction score required as the highest confidence (0.9).

We evaluated protein interactions among DEGs to obtain a view of their involvement in the development of IPF using STRING and Cytoscape software. The results indicated that the DEGs form a complex interaction network containing 1863 nodes and 3310 edges with an average node degree of 3.55 and a clustering coefficient of 0.33. The expected number of edges was 2430, which means that it was much smaller than the actual edges found, and the *p*-value of PPI enrichment was <1.0 × 10^−16^ (Figure 4, Table S4). Nodes not connected to the network were excluded. The network had considerably more interactions than expected, indicating that the DEGs are biologically connected as a group.

**Figure 5:**
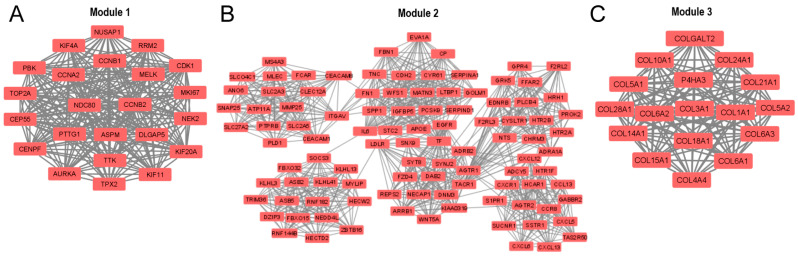
The three main modules of the PPI network were identified by the Cytoscape MCODE add-on: (**A**) module 1, (**B**) module 2, and (**C**) module 3. The default parameters were degree cutoff = 2, node score cutoff = 0.2, k-core = 2, and maximum depth = 100.

In addition, the entire PPI network was analyzed using the Molecular Complex Detection (MCODE) add-on of Cytoscape software, and 51 significant modules were identified. The three modules with the highest average MCODE score, module 1 (21,545), module 2 (17,660), and module 3 (16,875), contained 23, 101, and 17 genes, respectively, in addition to 237, 883, and 135 edges, respectively (Figure 5A–C). Each of these modules can be interpreted as significantly functional modules.

**Figure 6:**
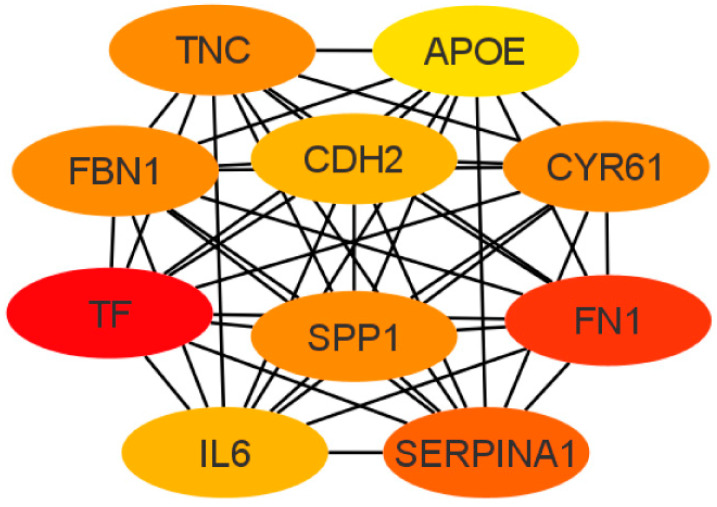
Top 10 hub gene networks. The top 10 hub genes derived from the MMC method were identified using the cytoHubba add-on.

Next, the cytoHubba add-on of Cytoscape software was used to identify the top 10 hub genes from DEGs by the maximal clique centrality (MCC) method, which were tenascin-c (*TNC*), cadherin 2 (*CDH2*), apolipoprotein E (*APOE*), secreted phosphoprotein 1 (*SPP1*), serpin family A member 1 (*SERPINA1*), fibrillin 1 (*FBN1*), interleukin 6 (*IL6*), fibronectin (*FN1*), cysteine-rich angiogenic inducer 61 (*CYR61*), and serotransferrin (*TF*) (Figure 6). Of these, seven represented upregulated genes (*CDH2*, *FBN1*, *FN1*, *APOE*, *SPP1*, *TF*, and *TNC*), and three represented downregulated genes (*CYR61*, *SERPINA1*, and *IL6*).

### 3.4. GO and KEGG Pathway Enrichment Analysis for the DEGs Present in the Three Main Modules of the PPI Network

We performed GO and KEGG analyses for the DEGs that are part of the three main modules present in the PPI network. BP was mainly enriched for extracellular matrix organization and the collagen catabolic process. MF was enriched for the structural constituent of the extracellular matrix and ubiquitin–protein transferase activity. Furthermore, CC was enriched for endoplasmic reticulum lumen and collagen trimers. The KEGG pathways enriched for the modules of which the DEGs are part are mainly related to ECM–receptor interaction, protein digestion and absorption, and neuroactive ligand-receptor interaction. The complete list of GO enrichment and KEGG pathway analyses can be found in Table S5.

### 3.5. GO Enrichment and KEGG Pathway Analyses for the Top 10 Hub Genes in the PPI Network

**Figure 7:**
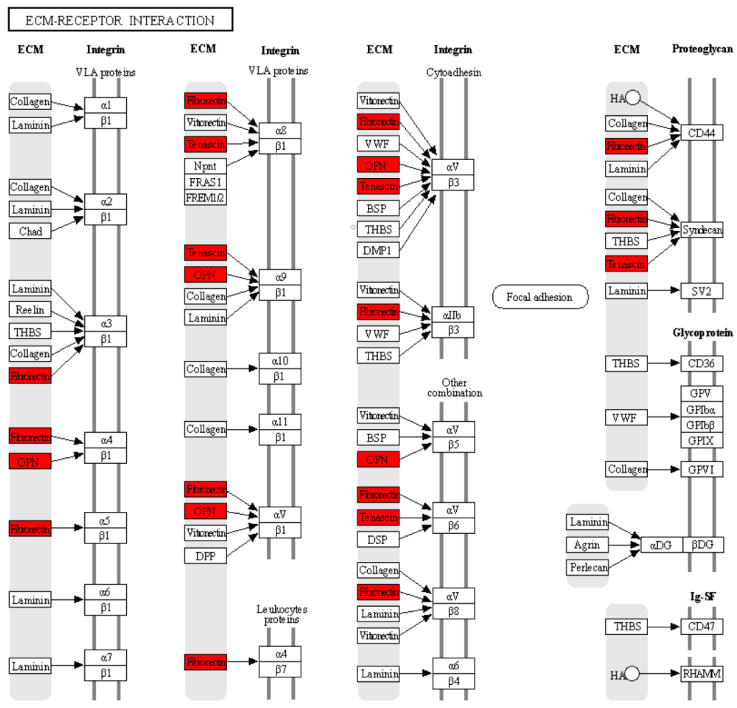
According to the KEGG analysis, the hub genes are related to the receptor–ECM interaction pathway. The red color indicates that three genes: SPP1 (OPN), TNC, and FN1 were significantly enriched in the receptor–ECM interaction pathway. The black arrows indicate the interaction of ECM proteins (OPN, TNC, and FN1) with cell surface proteins (integrins and proteoglycans).

To further understand the functions and pathways in which the 10 hub genes identified in the PPI network are involved, we performed GO enrichment and KEGG pathway analyses. The enriched GO terms were divided into BP, MF, and CC. The BP analysis showed that the hub genes were significantly enriched in extracellular matrix organization and osteoblast differentiation. For MF, the hub genes were enriched in integrin binding and heparin binding. In addition, relative to CC, the hub genes were enriched for the extracellular region and extracellular space. The results obtained from the KEGG pathway analysis indicated that three genes (*SPP1*, *TNC*, and *FN1*) were significantly enriched in the ECM–receptor interaction (Table 2). Within the ECM–interaction receptor pathway, we observe that SPP1, TNC, and FN1 play essential roles in the interaction between cells and the ECM. Together, these three proteins work in harmony with integrins, which act as major receptors on the cell membrane to facilitate cell adhesion, signaling, and cellular response in a variety of biological contexts and physiological processes. Their interaction in the ECM–interaction receptor pathway highlights their critical role in the regulation of events fundamental to cellular and tissue function, as shown in Figure 7.

### 3.6. In Silico Validation of Hub Genes

**Figure 8:**
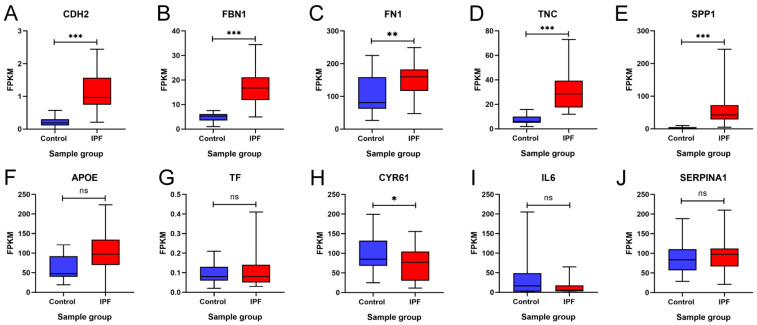
Validation of the expression of 10 hub genes in the GSE92592 dataset. Differences in the expression of the hub genes (**A**) *CDH2*, (**B**) *FBN1*, (**C**) *FN1*, (**D**) *TNC*, (**E**) *SPP1*, (**F**) *APOE*, (**G**) *TF*, (**H**) *CYR61*, (**I**) *IL6*, and (**J**) *SERPINA1* between the idiopathic pulmonary fibrosis (IPF) group and the control group are shown. FPKM = fragments per kilobase of transcript per million mapped reads. * = *p* < 0.05, ** = *p* < 0.01, and *** = *p* < 0.001. ns = not significant.

To validate our findings, these 10 hub genes were analyzed and validated in the GSE92592 dataset, which corresponds to a dataset obtained from mRNA sequencing of lung tissue from IPF and control samples. Figure 8 shows the expression profile of GSE92592 for the 10 hub genes. The results showed that *CDH2*, *FBN1*, *FN1*, *FN1*, *TNC*, *SPP1*, *APOE*, and *TF* maintained upregulation in IPF tissue samples compared to the control samples, and the increase was statistically significant except for *APOE* and *TF* (Figure 8A–G). On the other hand, the results showed that *CYR61* and *IL6* maintained downregulation in IPF tissue samples compared to the control samples; however, this decrease was not statistically significant for *IL6* (Figure 8H,I). In addition, the results showed that there was no statistically significant difference between the two groups for *SERPINA1* (Figure 8J). These results suggest that the expression profile of the GSE92592 dataset was consistent with the expression profile of the hub genes identified in our meta-analysis.

### 3.7. miRNA-mRNA Interaction Prediction and Network

**Figure 9:**
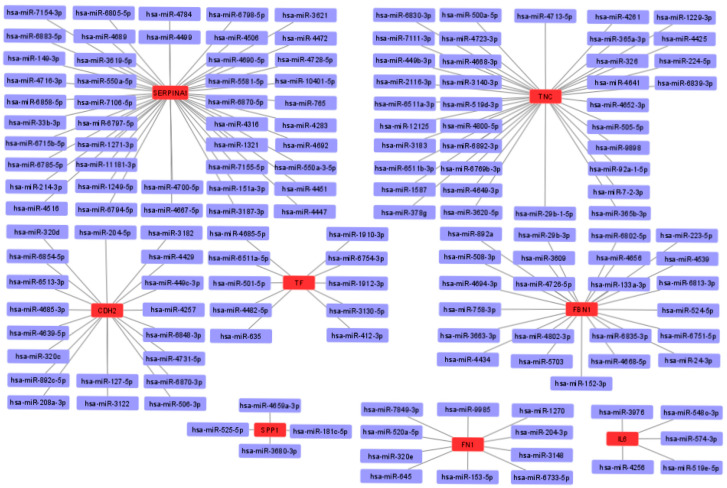
Interaction network between hub genes and their target miRNAs. Red-colored nodes represent hub genes, and blue-colored nodes are miRNAs.

To obtain possible miRNAs that regulated the expression of the hub genes, a miRWalk 3.0 database analysis was performed, and the results obtained indicated that 151 miRNAs are likely to target eight of the hub genes (*TNC*, *FBN1*, *CDH2*, *TF*, *SPP1*, *FN1*, *IL6*, and *SERPINA1*). In addition, no candidate miRNAs targeting the *APOE* and *CYR61* genes were identified. The miRNA-mRNA interaction network is shown in Figure 9. The results showed 44 miRNAs targeting *SERPINA1*, 36 miRNAs targeting *TNC*, 19 miRNAs targeting *CDH2*, 23 miRNAs targeting *FBN1*, 10 miRNAs targeting *TF*, 4 miRNAs targeting *SPP1*, 10 miRNAs targeting *FN1*, and 5 miRNAs targeting *IL6*.

### 3.8. Hub Genes in IPF Are Expressed in Lung Cancer and Are Associated with Cancer Progression

**Figure 10:**
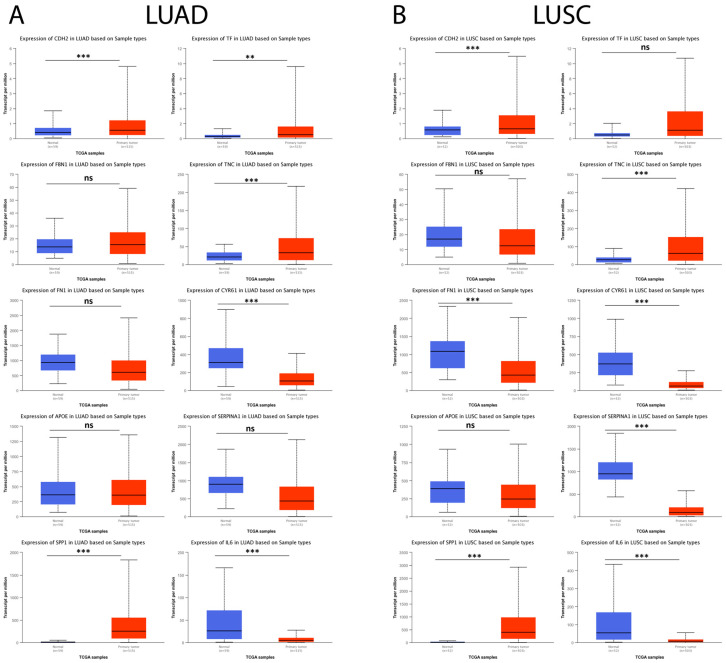
Expression levels of the 10 hub genes in lung cancer and normal tissue samples in the UALCAN database. (**A**) The expression levels of the 10 hub genes in LUAD patients and their respective controls. (**B**) The expression levels of the 10 hub genes in LUSC patients and their respective controls. ns = not significant, ** = *p* < 0.01, and *** = *p* < 0.001.

Subsequently, the possible relationship between hub genes identified in IPF and lung cancer (LUAD and LUSC) was explored using the UALCAN database. The results showed that, in LUAD tumor samples, *CDH2*, *SPP1*, *TF*, and *TNC* were significantly upregulated and *CYR61* and *IL6* were observed to be significantly downregulated compared to their respective controls, while, for *FBN1*, *FN1*, *APOE*, and *SERPINA1*, no statistically significant difference was observed between both comparison groups (Figure 10A). On the other hand, the results showed that, for LUSC tumor samples, *CDH2*, *SPP1*, and *TNC* were significantly upregulated and *FN1*, *CYR61*, *SERPINA1*, and *IL6* were significantly downregulated compared to their respective controls. In contrast, for *FBN1*, *APOE*, and *TF*, no statistically significant difference was observed between the comparison groups (Figure 10B).

**Figure 11:**
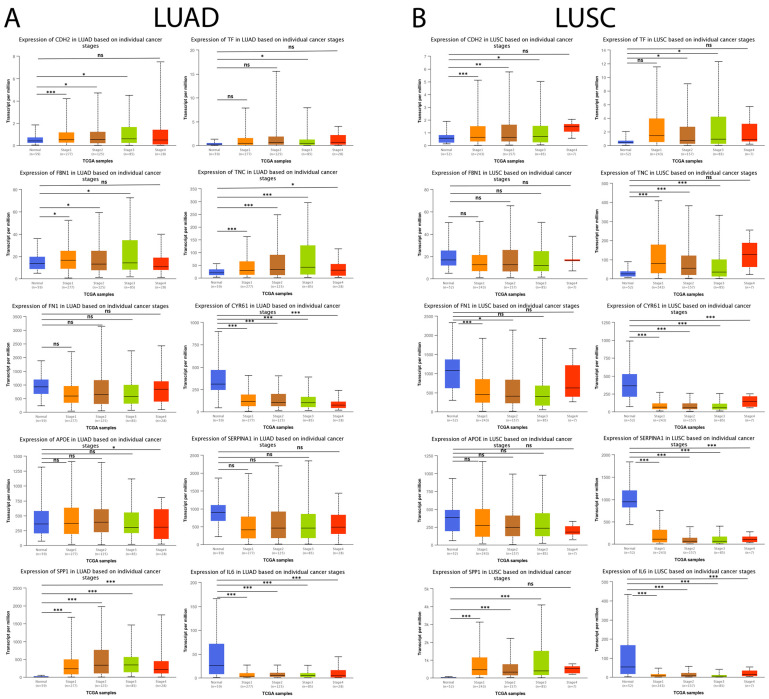
Expression levels of the 10 hub genes concerning individual cancer stages in lung cancer and normal tissue samples in the UALCAN database. (**A**) The expression level of the 10 hub genes in LUAD patients and their respective controls concerning individual cancer stages (stage 1, stage 2, stage 3, and stage 4). (**B**) The expression level of the 10 hub genes in LUAD patients and their respective controls concerning individual cancer stages (stage 1, stage 2, stage 3, and stage 4). ns = not significant, * = *p* < 0.05, ** = *p* < 0.01, and *** = *p* < 0.001.

Subsequently, we analyzed the expression level of the genes mentioned above concerning individual cancer stages (stage 1, stage 2, stage 3, and stage 4). As shown in Figure 11A, *CDH2*, *SPP1*, and *TNC* maintained a statistically significant increase associated with the progression of individual cancer stages in LUAD tumor samples compared to their respective controls. Furthermore, the results showed that the downregulation of *CYR61* and *IL6* expression maintained a statistically significant association with the progression of individual cancer stages in LUAD tumor samples compared to their respective controls. Moreover, the results obtained showed that *CDH2*, *SPP1*, and *TNC* maintained a statistically significant increase associated with the progression of individual cancer stages, and it was observed that the downregulation in *CYR61*, *SERPINA1*, and *IL6* expression maintained a statistically significant association with the progression of individual cancer stages in LUSC tumor samples compared to their respective controls (Figure 11B).

## 4. Discussion

In the present study, we identified hub genes and pathways involved in the development of IPF by a meta-analysis of five GEO microarray datasets with accession numbers GSE24206, GSE21369, GSE110147, GSE72073, and GSE32539. We revealed a total of 1888 DEGs, including 1105 upregulated and 783 downregulated DEGs. To better understand the functional levels of DEGs, we performed GO and KEGG enrichment analyses, and the results obtained showed that, for BP, upregulated genes were mainly enriched in cell adhesion, and downregulated genes were mainly enriched in angiogenesis. For MF, upregulated genes were mainly enriched in calcium ion binding, and downregulated genes were mainly enriched in protein kinase C activity. For CC, upregulated genes were mainly enriched in the proteinaceous extracellular matrix, and downregulated genes were mainly enriched in the plasma membrane integral component. Upregulated genes were significantly enriched in ECM–receptor interaction, protein digestion and uptake, and focal adhesion in the KEGG pathway enrichment analysis. In contrast, downregulated genes were significantly enriched in the steroid biosynthesis pathway, osteoclast differentiation, and the MAPK signaling pathway. These results correlate with those described in previous research that ECM–receptor interaction and focal adhesion are pathways that are extensively related to the development and progression of IPF, as these pathways have been described to regulate various biological processes such as proliferation, migration, and epithelial–mesenchymal transition (EMT) of resident lung fibroblasts, which triggers excessive ECM secretion and deposition, thus favoring the development of IPF [37,38].

Subsequently, our results showed the association between these DEGs and selected the three most significant modules of the PPI network according to the highest mean MCODE score. Finally, the top 10 hub genes with the highest degree were identified by the MCC method, including *TNC*, *CDH2*, *APOE*, *SPP1*, *SERPINA1*, *FBN1*, *IL6*, *FN1*, *CYR61*, and *TF*. In addition, we analyzed the top 10 hub genes by KEGG pathway enrichment analysis. The results obtained from the new study showed that three genes (*SPP1*, *TNC*, and *FN1*) were significantly enriched in ECM–receptor interaction, focal adhesion, and the PI3K-Akt signaling pathway. Among these genes, *TNC*, *FN1*, and *SSP1* have been studied and proposed as genes that play an essential role in the development and progression of IPF [39,40,41].

Our meta-analysis plays a critical role in improving the consistency and robustness of our results compared to individual microarray studies and other relevant gene expression investigations in IPF. By combining data from multiple microarray studies, we managed to significantly increase our sample size, providing a more robust and representative database. This, in turn, has improved the accuracy in identifying DEGs and increased the reliability of our results. Our results are supported by the consistency observed in other related studies that have explored gene expression in IPF using GEO datasets. By comparing our findings with previous research, we found a remarkable convergence in the expression of the core genes identified in our study with the results reported in other independent studies. This agreement strengthens the validity and biological relevance of our results. For example, some research focused on the bioinformatic analysis of genes, and pathways differentially expressed in IPF through the analysis of public databases identified *TNC*, *CDH2*, *FBN1* and *SPP1* as some of the main DEGs with the highest significant upregulation in samples from patients with IPF compared to their respective controls [16,42,43,44]. The results obtained from a study that analyzed the gene expression profiles of public databases of patients with acute exacerbation of IPF showed that *CYR61* is downregulated in patients with an acute exacerbation of IPF compared to patients who present stable fibrosis, which agrees with our results obtained for *CYR61* [45]. Furthermore, a study based on bioinformatic strategies identified eight genes significantly downregulated in samples from patients with IPF compared to their respective controls, among which *IL-6* was identified [46]. Similarly, another study focused on machine learning-based prediction of candidate gene biomarkers correlated with immune infiltration in IPF patients and identified that *IL-6* was significantly downregulated in IPF patient samples [44]. Additionally, a study that used two GEO datasets to establish and identify DEGs in IPF showed that *SERPINA1* was downregulated in IPF patients compared to the controls [47].

The *TNC* gene encodes the tenascin-c protein, a hexameric ECM glycoprotein that belongs to the tenascin family [39,48]. Physiologically, it is under strict regulation, being expressed mainly during embryogenesis and with practically undetectable expression in most adult tissues. Its transient expression has been associated with tissue injury and wound-healing processes [48,49]. Tenascin-c can exert different effects on many cell types and has a crucial role in modulating cell adhesion, proliferation, migration, angiogenesis, and innate and adaptive immunity [48,49]. The excessive and persistent accumulation of tenascin-c has been observed in various chronic pathological conditions, such as cancer and fibrosis [48,49,50]. Studies indicate that tenascin-c can induce the aberrant activation of lung fibroblasts and promote migration, EMT, and secretion of type I collagen on these cells [39,49]. Additionally, it was demonstrated by a bleomycin-induced murine model of IPF that *Tnc*-−/− mice manifest a significant reduction in the development of IPF [49,50]. Furthermore, studies indicate that tenascin-c is significantly elevated in the lung tissue of IPF patients, both at the gene and protein levels [39]. Therefore, we hypothesize that *TNC* may play a pivotal role in the pathogenesis of IPF.

The *SPP1* gene encodes a protein called secreted phosphoprotein 1, also known as osteopontin (OPN), a phosphorylated acidic glycoprotein initially detected in osteoblasts, and osteoclasts can bind to different ligands, such as integrins and fibronectin. In addition, it acts as a proinflammatory cytokine and has been implicated in various biological processes, such as the immune response, bone reconstruction, wound repair, adhesion, migration, and cell proliferation [51,52,53]. Additionally, it has been observed that *SPP1* mRNA is overexpressed in the lungs of IPF patients compared to healthy controls [52]. Likewise, different studies have shown that the SPP1 protein is overexpressed in the lung tissue, serum, and bronchoalveolar lavage (BAL) of IPF patients [52,53,54]. In vitro models have shown that SPP1 stimulation promotes cell proliferation, migration, and the adhesion of lung fibroblasts and alveolar epithelial cells (AECs) and favors increased ECM deposition [52,55]. Studies in murine models of bleomycin-induced IPF demonstrated that *Spp1*−/− mice are characterized by the development of pulmonary fibrosis due to cystic dilatation of the distal airways, accompanied by the reduced expression of type I collagen, TGF-β, and matrix metalloproteinase-2 compared to wild-type (WT) control mice; on the other hand, the administration of *Spp1* siRNA protects mice against bleomycin-induced pulmonary fibrosis [54,56]. Currently, SPP1 has been studied as a biomarker to diagnose IPF and monitor its progression. A study performed on a small cohort of 32 patients with acute exacerbation of IPF (AE-IPF), 39 patients with stable IPF (S-IPF), and 20 control subjects demonstrated that serum SPP1 concentrations in patients with AE-IPF significantly increased compared to the S-IPF patients or control subjects, suggesting that OPN is a potential biomarker for monitoring the onset of AE-IPF and a predictor of the survival of patients with IPF [53]. Thus, although the data on the role of OPN in the development of IPF and its potential utility as a diagnostic and prognostic biomarker for IPF are limited, they are encouraging and warrant future research on this molecule and its relationship to the pathogenesis of IPF.

The *FN1* gene encodes the fibronectin (FN) protein, a multifunctional glycoprotein that localizes to the ECM of different tissues and plasma [39]. Available reports have described two primary forms of FN: plasma FN that lacks the extra type III A (EDA) and extra type III B (EDB) sequences, which are secreted as a dimeric protein and produced mainly by hepatocytes, and cellular FN, which contains variable proportions of EDA and EDB, is a multimeric shaped protein present on the cell surface and is deposited in the ECM of different tissues, synthesized mainly by epithelial, mesenchymal, and inflammatory cells [39,57,58]. FN facilitates the vital connections of cells through its interaction with integrins and other receptors, which allows it to regulate different biological processes, such as cell adhesion, migration, and differentiation [57]. Recently, studies have shown that FN expression increases at the mRNA and protein levels in lung tissue undergoing a fibrotic process [59]. Furthermore, it has been observed that treatment of lung fibroblasts and AECs with TGF-β promotes an increase in *FN1* mRNA expression and FN protein production [60]. On the other hand, fibroblasts with a senescent phenotype associated with IPF were observed to secrete extracellular vesicles (EVs) that carry elevated levels of FN on their surface. These FN-enriched EVs stimulated an invasive phenotype in recipient fibroblasts by interacting with α5β1 integrin and promoting the activation of cell invasion-related signaling pathways involving mainly focal adhesion kinases (FAKs) and Src family kinases [41]. Additionally, it was demonstrated by a bleomycin-induced murine model of IPF that EDA−/− mice do not develop severe fibrosis compared with WT mice, suggesting that EDA-FN plays a pivotal role in the experiment-induced fibrogenesis process [58]. Therefore, these findings propose that the upregulation of *FN1* may play a critical role in developing IPF.

The *FBN1* gene encodes a fibrillin-1 protein, a multidomain extracellular glycoprotein that plays an essential role in maintaining the function and integrity of connective tissues [61,62]. Increased expression of fibrillin-1 has been associated with the development of fibrosis in organs such as the skin, liver, and kidneys [63,64,65]. It has been described that fibrillin-1 can interact directly with cell surface transmembrane receptors such as integrins and thus favor fibroblast proliferation [62]. On the other hand, recent studies have reported that microfibrils isolated from the skin of Tsk−/− mice, a model of systemic sclerosis, maintain a statistically significant increase in fibrillin-1 and are associated with the upregulation of a prooxidant phenotype in endothelial cells, thus facilitating their activation and mesenchymal transition [63]. However, despite its great biological importance, the role of fibrillin-1 in IPF has not yet been fully elucidated.

The *APOE* gene encoding the protein apolipoprotein E (ApoE), an essential lipoprotein in lipid metabolism, thus plays an essential role in the maintenance of plasma lipid homeostasis [66,67]. ApoE is mainly expressed in the liver; however, its expression has also been detected in the lungs, mainly in alveolar macrophages, pulmonary artery smooth muscle cells (PASMCs), and AEC type I and type II [67]. Several investigations have shown that ApoE plays a significant role in pulmonary homeostasis and the pathogenesis of multiple respiratory diseases through its ability to attenuate inflammation, oxidative stress, and tissue remodeling responses [67]. For example, ApoE−/− mice stimulated with inhaled lipopolysaccharide (LPS) or subjected to direct airway inoculation with CXCL1 have shown increased neutrophil and monocyte recruitment into the airways compared to their respective controls. In contrast, ApoE mimetic peptide (COG1410) treatment significantly reduced airway neutrophilia [68]. In addition, a study performed in a bleomycin-induced IPF model on ApoE−/− and WT mice provided evidence that ApoE plays a beneficial role in facilitating fibrosis resolution, and the mice were evaluated eight weeks after treatment with saline or bleomycin, a period in which the resolution of fibrosis in bleomycin-treated lungs has been observed. At the time of evaluation, the degree of fibrosis in the lungs of bleomycin-treated ApoE−/− mice was evident throughout the resolution phase, as evidenced by significantly increased pulmonary hydroxyproline, pulmonary collagen deposition, and expression of profibrotic mediators compared to WT mice [69]. These results suggest that ApoE plays an essential role in the regulation of fibrogenic processes associated with IPF.

The *CDH2* or cadherin-2 gene encodes the N-cadherin protein, a transmembrane glycoprotein and a vital member of the cadherin family, a class of molecules that plays an essential role in cell-cell adhesion [70,71]. N-cadherin is widely expressed in embryos and is actively involved in developing and regulating nervous tissue, heart, brain, skeletal muscles, blood vessels, and other organs [71]. However, recent research has shown that N-cadherin is aberrantly expressed in various cancer types [70,71,72]. During EMT, there is a switch from E-cadherin (downregulation) to N-cadherin (upregulation), which results in the loss of epithelial integrity, thus promoting the migratory and invasive capacity of cancer cells and favoring tumor development [71,72]. EMT is a critical factor in the development of IPF; during this process, a significant increase has been observed in myofibroblasts derived from epithelial cells that show a significant increase in N-cadherin and α-smooth muscle actin (α-SMA) and a decrease in E-cadherin expression as a result of EMT [73,74,75]. Importantly, these myofibroblasts are critical players in developing IPF, because they maintain high-rate proliferation, invasion, migration, and excessive aberrant ECM production [73,74]. Therefore, the inhibition of EMT with various drugs has been widely studied for its beneficial effects in sufficiently ameliorating pulmonary fibrosis [75,76].

The *TF* gene encodes the transferrin protein, a glycoprotein that plays an essential role as an iron transport protein in the blood and is, therefore, a key player in iron metabolism [77]. Iron (Fe) is a metal and an essential nutrient for cells and is necessary for cellular processes such as oxygen transport, oxidative phosphorylation, immune function, and DNA synthesis [77,78]. In addition, the association of increased iron concentrations and imbalance in the metabolism of this nutrient with the development of some types of cancer and lung diseases has recently been studied [77,78,79]. Recent studies have shown that iron levels are upregulated in the alveolar epithelial lining fluid of IPF patients compared to controls [80]. Another study demonstrated that bronchoalveolar lavage (BAL) of patients with acute respiratory distress syndrome (ARDS) maintains higher concentrations of total and nonheme iron compared to healthy controls. Furthermore, this increase in iron was associated with an increase in iron metabolism-related proteins such as transferrin, hemoglobin, TfR1, lactoferrin, and ferritin in BAL patients with ARDS [81]. Thus, the available evidence suggests that an imbalance in iron concentrations and iron-related proteins such as transferrin may participate in the fibrogenic process of IPF, an intriguing phenomenon that needs to be addressed in future studies.

The *SERPINA1* gene encodes alpha-1 antitrypsin (AAT), a glycoprotein produced mainly in the liver by hepatocytes, which exerts an essential anti-inflammatory function due to its ability to inhibit serine proteases, mainly neutrophil elastase. AAT plays an essential role in protecting alveolar tissue from proteolytic damage produced mainly by neutrophil elastase [82,83]. Alpha-1 antitrypsin deficiency (AATD) is an inherited genetic disorder; currently, approximately 125 genetic polymorphisms of the *SERPINA1* gene have been described as associated with the development of lung diseases such as chronic obstructive pulmonary disease (COPD), pulmonary fibrosis, and lung cancer [83,84]. A systematic review including six studies and a total of 4038 lung cancer patients showed that AATD might increase the risk of developing lung cancer [83]. Furthermore, although the association between AATD and pulmonary fibrosis is infrequent, this correlation has been described in some studies and case reports, which has generated an important debate on the role of AAT in the pathogenesis of pulmonary fibrosis. [82,84]. Therefore, it is essential to emphasize that further studies are needed to better understand whether AATD plays an essential role in the development of pulmonary fibrosis.

The *CYR61* gene encodes the cysteine-rich angiogenic inducer 61 protein, currently referred to as connective tissue growth factor (CCN1), which is a matrix protein belonging to the CCN family [85,86]. CCN1 binds to matrix proteins such as heparan sulfate and glycosaminoglycans and can interact with transmembrane receptors on the cell surface, such as integrins [86,87]. Therefore, CCN1 is involved in various cellular processes, such as cell adhesion, proliferation, migration, growth, differentiation, apoptosis, and cellular senescence [85,86]. Recent research has shown that CCN1 is involved in the pathological processes of diseases such as fibrosis and cancer [88,89]. In addition, CCN1 has been shown to play a role in the development of various lung diseases, such as pulmonary fibrosis [85]. However, the data obtained from various studies have generated much debate regarding the profibrotic or antifibrotic effects that CCN1 may exert on the development of pulmonary fibrosis [90,91]. For example, it was demonstrated that TGF-β1, a profibrotic cytokine, induces CCN1 expression on lung fibroblasts and that siRNA-mediated CCN1 silencing significantly attenuated the TGF-β1-mediated induction of fibrotic proteins such as Col1a1, Col1a2, FN, and α-SMA. In addition, it was also demonstrated that siRNA-mediated CCN1 silencing significantly attenuated bleomycin-induced lung injury in a murine model [91]. Moreover, the available evidence indicates that CCN1 may also exert an antifibrotic effect by inducing the senescence and apoptosis of fibroblasts and myofibroblasts. In addition, CCN1 was shown to promote senescence by inducing the DNA damage response, reactive oxygen species (ROS) generation, and p53 and p16 activation [90]. The evaluation of CCN1 expression in the plasma of patients with IPF reported that the median survival time was 3.3 years for patients with high CCN1 levels (≥0.147 ng/mL) and 5.7 years for patients with low CCN1 levels (<0.147 ng/mL), suggesting that patients with high plasma CCN1 levels had a nearly two-fold increased risk of death compared to subjects with low plasma CCN1 levels [92]. Therefore, our results encourage further elucidation of this intriguing proposition because of the essential role of CCN1 in cellular senescence and the development of IPF.

The *IL6* gene encodes the protein interleukin-6 (IL-6), a cytokine with multiple functions associated with immune responses and inflammation; therefore, it has been closely related to the pathology of different chronic inflammatory and autoimmune diseases [93,94]. Recent research has shown that IL-6 is actively involved in various inflammatory processes associated with the pathogenesis of various chronic lung diseases [95]. In addition, IL-6 is elevated in murine models of silica- and bleomycin-induced pulmonary fibrosis and in humans with pulmonary fibrosis [95,96,97]. Studies in a murine model of bleomycin-induced IPF have shown that IL-6 −/− mice exhibit significant attenuation in the development of lung inflammation and fibrosis compared to WT mice [98]. Similarly, an in vivo blockade of IL-6 signaling using recombinant gp130Fc, a selective inhibitor, was shown to maintain a positive effect on reducing bleomycin-induced lung inflammation and fibrosis in a murine model and was accompanied by a marked improvement in respiratory function [99]. However, in vitro studies have demonstrated that IL-6 plays an antioxidant role in reducing ROS-induced alveolar epithelial type II cell death [100]. These data suggest that IL-6 may play a bidirectional role in the pathogenesis of pulmonary fibrosis. However, its effect on pulmonary fibrosis and the mechanisms associated with this disease remain an unmet need. Furthermore, one of the most notable observations in our study is the discrepancy in the results of IL6 expression compared to some previous studies that have reported its overexpression in IPF. Our meta-analysis revealed that IL6 remained downregulated in IPF tissue samples compared to the control samples, although this decrease did not reach statistical significance according to the results obtained from validation of the GSE92592 dataset. The absence of statistical significance may be related to the sample size and the large biological variability between patients with IPF, which makes it difficult to detect significant differences in a smaller dataset [101,102]. Despite this discrepancy with the literature on IL-6 expression, we consider our findings valuable and enriching for the field of IPF research. The identification of other DEGs and hub genes in our study supports the biological importance of these genes in IPF, even if IL6 did not show significant regulation in our dataset. This discrepancy underscores the need for further research to better understand IL6 expression in IPF and its role in disease pathogenesis. Future studies could consider subgroups of patients or confounding factors that may influence IL6 expression. Furthermore, performing additional functional studies could provide a more complete view of the role of IL6 in IPF.

Recently, miRNAs have been extensively studied for their essential role in regulating gene expression at the posttranscriptional level and their relationship with various biological processes [103]. It has been shown that miRNAs play a critical role in developing and progressing multiple lung diseases, such as IPF [103,104]. miR-130a-3p has been shown to participate in the regulation of IPF by inhibiting lung fibroblast differentiation by blocking the activation of the TGF-β/Smad signaling pathway [104]. Additionally, it has been observed that miR-199a-5p is upregulated in patients with IPF. This miRNA acts as an effector of TGF-β signaling in lung fibroblasts, stimulating their proliferation, migration, invasion, and differentiation into myofibroblasts [105].

For this reason, we used the miRWalk 3.0 database to predict miRNAs that could target the hub genes identified in our study. A total of 151 miRNAs targeted eight hub genes (*TNC*, *FBN1*, *CDH2*, *TF*, *SPP1*, *FN1*, *IL6*, and *SERPINA1*). Interestingly, some of these miRNAs have been studied for their involvement in the progression of IPF and other lung diseases. For example, it has been shown that miRNA-326 maintains decreased expression during the development of IPF. It has been shown that this miRNA negatively regulates the expression of TGF-β and other profibrotic genes such as *COL1A2*, *COL3A1*, and *SMAD3*. In addition, it promotes the upregulation of antifibrotic genes such as *IL10* and *SMAD7* [106].

On the other hand, a study showed that miR-320c downregulates *SERPINA1* expression. This miRNA could be a biomarker of inflammation in lung diseases, because its blood levels are elevated in patients with emphysema, bronchiectasis, chronic bronchitis, and asthma [82]. Therefore, we hypothesized that these miRNAs might be involved in different molecular processes related to IPF development and progression. Furthermore, the miRNA–miRNA predictions provided in our study are a starting point for future experimental investigations that could shed light on the molecular mechanisms underlying IPF and its relationship with lung cancer and thus contribute to the advancement in the understanding and treatment of these diseases.

Lung cancer ranks second among the most commonly diagnosed cases and is the leading cause of cancer death worldwide, and lung cancer incidence and mortality are estimated to increase dramatically within the next few years [107]. The available evidence suggests that lung cancer is one of the main complications of patients with IPF [15]. Therefore, we explored whether these ten hub genes identified in IPF were associated with progression in LUAD and LUSC, two of the most frequent types of lung cancer in patients with IPF [15]. Interestingly, *CDH2*, *SPP1*, and *TNC* were significantly upregulated, and *CYR61*, *SERPINA1*, and *IL6* were significantly downregulated in LUAD and LUSC tumors compared to the normal samples. Interestingly, we also observed that the expression of *CDH2*, *SPP1*, *TNC*, *CYR61*, *SERPINA1*, and *IL6* correlated with the progression of individual cancer stages. Surprisingly, the available evidence suggests that *CDH2* [108,109], *SPP1* [110], *TNC* [111], *CYR61* [85], *SERPINA1* [83,112], and *IL6* [113] play an essential role in the development of lung cancer; therefore, our results suggest that these genes might exert central roles in the pathogenesis of IPF and are excellent targets to study the development, progression, and prognosis of IPF-associated lung cancer.

In the present study, we identified hub genes that could be potential biomarkers for diagnosing IPF. Likewise, these hub genes could be further investigated for their involvement in the pathogenesis of IPF. However, the present study contains some limitations, such as the requirement of additional experiments to complement the results obtained in the bioinformatics analysis. Therefore, it is suggested to consider further studies to evaluate the association between these hub genes and IPF.

## 5. Conclusions

In conclusion, a meta-analysis of microarray-generated gene expression datasets from lung tissue of IPF patients and healthy controls provided a profile of DEGs that may be involved in developing IPF. This hub gene profile (*TNC*, *CDH2*, *APOE*, *SPP1*, *SERPINA1*, *FBN1*, *IL6*, *FN1*, *CYR61*, and *TF*) can be considered an important target to investigate its relationship with the molecular mechanisms associated with IPF development and to evaluate it as a candidate diagnostic and prognostic biomarker. Finally, we evaluated the expression of hub genes with the development of one of the main complications of IPF patients, LUAD and LUSC, and observed that six of them (*CDH2*, *SPP1*, *TNC*, *CYR61*, *SERPINA1*, and *IL6*) were correlated with the progression of different cancer stages. Thus, we provide valuable and novel information on potential candidate genes to study the progression and prognosis of IPF-associated lung cancer. However, further confirmation by a series of molecular biology experiments is required to confirm the functions of the hub genes identified in this bioinformatics analysis.

## Figures and Tables

**Table 1:** Overview of the datasets included in the meta-analysis.

GEO ID	Platform	Samples		Reference
		Control	IPF	
GSE24206	GPL570	6	17	[19]
GSE21369	GPL570	6	11	[20]
GSE110147	GLP6244	11	22	[21]
GSE72073	GPL6480	3	5	[22]
GSE32539	GPL6244	50	119	[23]
Total		76	174	

**Table 2:** Most significant terms for each of the three categories of GO analysis and KEGG pathway analysis for the 10 hub genes identified in the PPI network.

Category	Term	Count	*p*-Value	Genes
BP	GO:0030198~extracellular matrix organization	5	2.17 × 10^−6^	*SPP1*, *TNC*, *FN1*, *CYR61*, *FBN1*
BP	GO:0007155~cell adhesion	5	6.23 × 10^−5^	*CDH2*, *SPP1*, *TNC*, *FN1*, *CYR61*
BP	GO:0006953~acute-phase response	3	1.87 × 10^−4^	*IL6*, *SERPINA1*, *FN1*
BP	GO:0022617~extracellular matrix disassembly	3	7.13 × 10^−4^	*SPP1*, *FN1*, *FBN1*
BP	GO:0042060~wound healing	3	7.90 × 10^−4^	*IL6*, *TNC*, *FN1*
MF	GO:0008201~heparin binding	4	6.73 × 10^−5^	*FN1*, *APOE*, *CYR61*, *FBN1*
MF	GO:0005178~integrin binding	3	0.00134085	*FN1*, *CYR61*, *FBN1*
MF	GO:0050840~extracellular matrix binding	2	0.01377989	*SPP1*, *CYR61*
CC	GO:0005576~extracellular region	9	3.03 × 10^−8^	*IL6*, *TF*, *SERPINA1*, *SPP1*, *TNC*, *FN1*, *APOE*, *CYR61*, *FBN1*
CC	GO:0005615~extracellular space	8	3.74 × 10^−7^	*IL6*, *TF*, *SERPINA1*, *SPP1*, *TNC*, *FN1*, *APOE*, *FBN1*
CC	GO:0031012~extracellular matrix	5	8.06 × 10^−6^	*TNC*, *FN1*, *APOE*, *CYR61*, *FBN1*
CC	GO:0005578~proteinaceous extracellular matrix	4	2.47 × 10^−4^	*SERPINA1*, *FN1*, *CYR61*, *FBN1*
CC	GO:0070062~extracellular exosome	7	7.38 × 10^−4^	*TF*, *SERPINA1*, *CDH2*, *SPP1*, *FN1*, *APOE*, *FBN1*
KEGG	hsa04512: ECM–receptor interaction	3	0.00318652	*SPP1*, *TNC*, *FN1*
KEGG	hsa04151: PI3K-Akt signaling pathway	4	0.00376323	*IL6*, *SPP1*, *TNC*, *FN1*
KEGG	hsa04510: Focal adhesion	3	0.01697039	*SPP1*, *TNC*, *FN1*

## Data Availability

Publicly available microarray datasets were analyzed in this study. These microarray datasets can be found in the GEO database (https://www.ncbi.nlm.nih.gov/geo/, accessed on 2 September 2022) with accession numbers GSE24206, GSE21369, GSE110147, GSE72073, and GSE32539. All data generated or analyzed during this study are included in this published article and its supplementary information files.

## Supplementary Materials

The following supporting information can be downloaded at https://www.mdpi.com/article/10.3390/arm91050032/s1: Table S1. Complete list of differentially expressed genes (DEGs). Table S2. Gene Ontology enrichment and KEGG pathway analyses of significantly upregulated genes. Table S3. Gene Ontology enrichment and KEGG pathway analyses of significantly downregulated genes. Table S4. PPI network in tabular form. Table S5. Gene Ontology enrichment and KEGG pathway analyses of DEGs are present in the three main modules of the PPI network.
